# Can Enhancing Neuronal Activity Improve Myelin Repair in Multiple Sclerosis?

**DOI:** 10.3389/fncel.2021.645240

**Published:** 2021-02-23

**Authors:** Dorien A. Maas, María Cecilia Angulo

**Affiliations:** ^1^Université de Paris, Institute of Psychiatry and Neuroscience of Paris (IPNP), INSERM U1266, Paris, France; ^2^GHU PARIS Psychiatrie et Neurosciences, Paris, France

**Keywords:** remyelination, neuronal activation, non-invasive brain stimulation, oligodendrocyte (OL) lineage cells, multiple sclerosis, adaptive myelination, neuron-oligodendroglia interactions

## Abstract

Enhanced neuronal activity in the healthy brain can induce *de novo* myelination and behavioral changes. As neuronal activity can be achieved using non-invasive measures, it may be of interest to utilize the innate ability of neuronal activity to instruct myelination as a novel strategy for myelin repair in demyelinating disorders such as multiple sclerosis (MS). Preclinical studies indicate that stimulation of neuronal activity in demyelinated lesions indeed has the potential to improve remyelination and that the stimulation paradigm is an important determinant of success. However, future studies will need to reveal the most efficient stimulation protocols as well as the biological mechanisms implicated. Nonetheless, clinical studies have already explored non-invasive brain stimulation as an attractive therapeutic approach that ameliorates MS symptomatology. However, whether symptom improvement is due to improved myelin repair remains unclear. In this mini-review, we discuss the neurobiological basis and potential of enhancing neuronal activity as a novel therapeutic approach in MS.

## Introduction

“Neuronal activity-dependent” or “adaptive” myelination is the process by which electrical activity of neuronal axons instructs oligodendroglial cells to myelinate these active axons, thereby increasing their conduction velocity. Increased myelination is often associated with improved brain function and this makes activity-driven myelination an attractive way to modulate neuronal circuit function and behavior. For example, learning to play the piano increases white matter integrity in the human brain, a measure associated with changes in white matter microstructures that include alterations of myelin sheaths (Han et al., 2009). Similarly, learning a complex motor task leads to increased myelination in motor brain areas in the mouse, while when this *de novo* myelination was blocked, motor learning did not occur to the same extent (McKenzie et al., 2014). Moreover, myelination defects of cortical parvalbumin interneurons results in a decreased inhibition and an excitation/inhibition imbalance that correlates with whisker-dependent texture discrimination impairments (Benamer et al., 2020). Neuronal activity-dependent myelination in animal models can be achieved in various ways including direct neuronal stimulation by optogenetics or pharmacogenetics as well as indirect neuronal stimulation using behavioral paradigms or non-invasive brain stimulation like transcranial magnetic stimulation (TMS) and transcranial direct current stimulation (tDCS). The fact that neuronal activity can achieve *de novo* myelination in the healthy brain leads to the hypothesis that this is an innate biological mechanism that could be used to promote myelin regeneration under pathological conditions.

**FIGURE 1:**
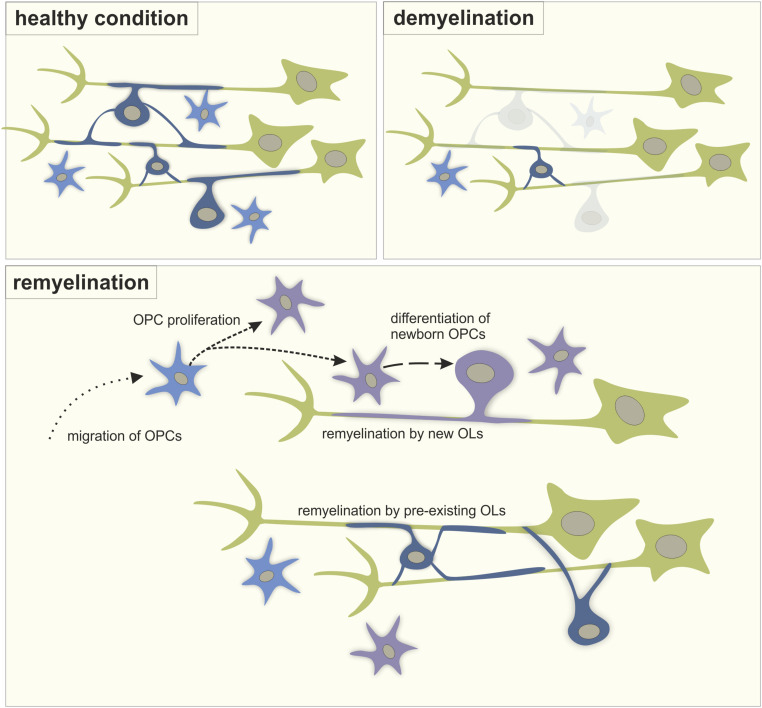
The process of remyelination. A schematic representation of myelination in healthy condition, during demyelination and during the process of remyelination involving the migration of OPCs into the lesion from other brain regions, the proliferation of OPCs, the differentiation of new OPCs into myelinating OLs and the remyelination of naked axons by both newly differentiated OLs and pre-existing OLs. Green: neurons, light blue: OPCs, dark blue: mature OLs, purple: new-born cells.

Despite the limited regenerative capacity of the CNS, myelin repair, or remyelination, can occur spontaneously in demyelinating diseases such as multiple sclerosis (MS). The most accepted mechanism of remyelination consists of both recruitment and proliferation of oligodendrocyte precursor cells (OPCs) as well as the differentiation of these new OPCs into mature myelinating oligodendrocytes (OLs) (Figure 1). However, recent studies in humans and rodents also support the idea that pre-existing OLs are a major source of myelin sheath regeneration (Figure 1; Jäkel et al., 2019; Yeung et al., 2019; Bacmeister et al., 2020). While remyelination is essential to prevent degradation of naked axons and restore axon potential conduction in neurons in the demyelinated lesion, remyelination failure is common in MS. Current therapies, mainly focused on the inflammatory component of the disease, are insufficient to prevent the consecutive neuronal loss that causes disabilities. Although recent clinical trials have tested the pro-remyelinating potential of several molecules, treatment strategies that effectively restore myelin in demyelinated lesions still need to be developed (Lubetzki et al., 2020). In this context, enhancing neuronal activity represents an attractive alternative strategy for remyelination in brain disorders where myelination is affected. In this minireview, we will address the potential of neuronal activity-enhanced remyelination as a novel therapeutic strategy in MS.

## Neuronal Activity-Induced Myelination in the Healthy Brain

Initial studies in the 90’s demonstrated that neurons communicate with OL lineage cells at different stages of OL maturation, thereby modulating the myelination process (Barres and Raff, 1993; Demerens et al., 1996). From recent studies on adaptive myelination *in vivo*, it became clear that activation of neurons can stimulate the proliferation of OPCs as well as their differentiation into myelinating OLs. For example, optogenetic stimulation of layer V projection neurons leads to increased OPC proliferation and differentiation and an increase in myelin thickness (Gibson et al., 2014). Moreover, neuronal activity improved motor function. The use of DREADDs to pharmacogenetically enhance neuronal activity of somatosensory neurons in mice also leads to increased proliferation and differentiation of OPCs as well as a strong bias for newly differentiated OLs to myelinate activated axons (Mitew et al., 2018). When parvalbumin interneurons of the medial prefrontal cortex (mPFC) are activated using DREADDs, a significant increase in total length of myelination occurs (Stedehouder et al., 2018). Notably, increasing cortical activity by silencing parvalbumin interneurons using optogenetics in the anterior cingulate cortex results in an enhanced proliferation of OPCs, while decreasing cortical activity by optogenetically activating parvalbumin interneurons leads to decreased proliferation of OPCs (Piscopo et al., 2018). In this study, the number of newly generated myelinating OLs was increased in mice with increased cortical activity, but not altered in mice with reduced cortical activity. In line with this, activating parvalbumin interneurons with DREADDs, which should reduce the activity of neuronal networks, did not affect the number of myelinating OLs in the mPFC (Stedehouder et al., 2018). Taken together, these studies indicate that active axons receive increased myelination regardless of neuronal identity. They also suggest that the effect of neuronal activity on OPC proliferation and differentiation likely depends on the pattern of activity within neuronal networks. This is supported by a study showing that neuronal stimulation at various frequencies *in vivo* has different effects on OPC proliferation and differentiation (Nagy et al., 2017). Indirect measures of neuronal activation can also influence OLs and myelination. It has been demonstrated in mice that 14 or 28 days of repetitive TMS increased the survival of new OLs leading to a larger number of myelinating OLs and increased myelin internode length in the cortex (Cullen et al., 2019). This is interesting because TMS is a method with unknown biological mechanisms that has already been tested as a therapy for MS in clinical studies (Centonze et al., 2007; Hulst et al., 2017). It should be noted that the benefits of neuronal activity may not be solely attributed to effects on OL lineage cells and remyelination as neuronal activity is also known to affect neurons, astrocytes and microglial cells (Li et al., 2012; Hasel et al., 2017). As such, it remains unclear to what extent remyelination alone is necessary for the functional benefits of enhanced neuronal activity.

How electrically active axons communicate with OPCs and OLs to promote myelination remains unclear. However, there are multiple synaptic and non-synaptic signaling mechanisms that may play a role (Hines et al., 2015; Mensch et al., 2015; Wake et al., 2015; Hughes and Appel, 2019). Electrically active axons release potassium which depolarizes OPCs and leads to the opening of voltage-gated calcium channels on OPCs (Barron and Kim, 2019). Activated, unmyelinated axons release glutamate (Kukley et al., 2007; Ziskin et al., 2007) which activates AMPA (Barron and Kim, 2019), NMDA (Doyle et al., 2018) as well as metabotropic glutamate receptors (Wake et al., 2015) on oligodendroglia. Neuronal activity can also activate GABA and muscarinic receptors on OPCs (Paez and Lyons, 2020). In addition, purinergic receptors on oligodendroglia can be activated by activity-induced increases of extracellular ATP (Welsh and Kucenas, 2018). The activation of the above-mentioned receptors and transporters converges on an increase in the intracellular calcium level in oligodendroglia. Accordingly, calcium signaling in oligodendroglia increases OPC proliferation (Ohashi et al., 2018), OPC morphological maturation (Waggener et al., 2013), OL differentiation (Cheli et al., 2018), the expression of myelin-related transcription factors (Weider et al., 2018) and proteins (Cheli et al., 2018), as well as myelin sheath thickness (Waggener et al., 2013). Moreover, the frequency and amplitude of the calcium signals in OLs determine whether myelin sheaths grow or retract (Baraban et al., 2018; Krasnow et al., 2018). Alternatively, it has been proposed that activity-dependent myelination is mediated by the morphology of the to-be-myelinated axon (Stedehouder et al., 2019) or *via* mechanotransduction (Lourenço and Grãos, 2016). Taken together, in the healthy brain, communication between electrically active neurons and oligodendroglial cells affects OL proliferation and differentiation, and can initiate *de novo* myelination resulting in behavioral changes. Consequently, this makes stimulation of neuronal activity an interesting approach for myelin repair in demyelinating disorders such as MS.

## Modulation of Neuronal Activity During Remyelination

While activity-driven myelination is accepted as a key mechanism in the CNS, literature on the effect of neuronal activity on myelin repair is still scarce. Nevertheless, a limited number of studies have reported on the potency of manipulating neuronal activity as a remyelination strategy in animal models. The intracerebral infusion of AMPA/kainate receptor antagonists and a voltage-dependent Na^+^ channel blocker in demyelinated lesions induced by ethidium bromide showed a decreased differentiation of OPCs into myelinating OLs, an increased OL apoptosis and a reduced remyelination (Gautier et al., 2015). These results suggest that blocking neuronal activity negatively affects remyelination. The reverse experiment was also initially proposed in a model of contusive spinal cord injury in rats, associated with demyelination without axonal loss (Li et al., 2017). Several days of electrical stimulation in the motor cortex increased the number of OPCs and newly myelinating OLs as well as enhanced remyelination in the remote demyelinated region, the spinal cord. However, it was only recently that a positive effect of neuronal activity on remyelination was unequivocally demonstrated *in vivo* (Ortiz et al., 2019). Distinct neural stimulation paradigms applied directly at the injured site had particular effects on remyelination. On the one hand, in lysolecithin-induced demyelinated lesions of the mouse corpus callosum, a single 3 h session of 20 Hz optogenetic stimulation of callosal axons led to increased proliferation of OPCs in the lesion the day of the stimulation. However, this new pool of progenitors did not result in a larger number of myelinating OLs 1 week later. On the other hand, a 3 h 20 Hz optogenetic stimulation session every day during 1 week increased OL differentiation, enhanced the number of remyelinated axons and improved action potential conduction in the lesion (Ortiz et al., 2019). This study using direct neuronal stimulation indicates that neuronal activity may indeed promote remyelination in demyelinated lesions under specific stimulation paradigms.

Recent studies in animal models have addressed the effects of indirect methods of neuronal stimulation on remyelination. As indirect neuronal stimulation methods such as TMS, tDCS and behavioral training are readily applicable in the clinic, it is worthwhile to investigate different stimulation methods in rodents. Sixty Hz electromagnetic stimulation increases remyelination in toxin demyelinated lesions of the rat corpus callosum (Sherafat et al., 2012) and low frequency electromagnetic stimulation promotes the differentiation of OPCs and remyelination after spinal cord injury in rats (Li et al., 2019). Epidural electrical stimulation after spinal cord injury also enhanced remyelination and increased OL differentiation and survival (Li et al., 2020). Likewise, brain activation using 20 Hz ultrasound bursts increased remyelination after cuprizone demyelination in mice (Olmstead et al., 2018) and voluntary exercise immediately after lysolecithin lesions of the spinal cord white matter increased OPC proliferation, differentiation and remyelination (Jensen et al., 2018). Notably, pairing voluntary exercise with the pro-myelinating drug clemastine showed an additive effect in increasing myelin repair (Jensen et al., 2018). Although it is likely that in both studies utilizing physical exercise neuronal activity in the demyelinated lesion was enhanced, neither study provides evidence that this was indeed the case. Nevertheless, the results of OL differentiation and more efficient remyelination are in line with the observations made in studies employing direct neuronal stimulation. In addition to the above-mentioned studies, it is known that brain stimulation can influence myelination and oligodendroglial dynamics after brain damage or traumatic injury. For example, tDCS in rats after focal cerebral ischemia lead to a faster recovery of motor function and stimulated the migration of OPCs to the lesion (Braun et al., 2016). Moreover, oscillating field stimulation leads to increased OPC differentiation and enhances functional recovery after spinal cord injury in rats (Jing et al., 2015). This was confirmed in a second study where enhanced remyelination and increased protein expression of oligodendroglial marker Olig2 were found (Zhang et al., 2014). Collectively, indirect methods of neuronal activation can positively influence myelin repair.

Behavioral paradigms recruiting remyelinating brain regions might be able to accelerate the remyelination process, likely through increased neuronal activity. A recent study reported increased neuronal firing rates in the motor cortex following cuprizone demyelination that recovered to normal during the same period in which remyelination was completed, suggesting a correlation between the level of neuronal activity and remyelination (Bacmeister et al., 2020). Behavioral training that recruits the motor cortex during remyelination increased the number of myelinating OLs and the number of myelin sheaths produced by both pre-existing and newly formed OLs relative to untrained demyelinated mice (Bacmeister et al., 2020). Behavioral experiences can also improve myelin recovery in other forms of brain injury. For example, increased OPC numbers were found in ischemic lesions after environmental enrichment (Komitova et al., 2006). After both environmental enrichment and behavioral skilled reaching training, the number of OPCs was increased in a rat model for stroke (Keiner et al., 2008). Likewise, environmental enrichment increased the number of OPCs in a rat model with hypomyelination (Maas et al., 2020) and increased the OL differentiation and myelination in a model of perinatal brain injury (Forbes et al., 2020).

Although neuronal activity can be enhanced in a controlled manner to limit deleterious effects such as excitotoxicity, it cannot be excluded that apart from benefits for remyelination, enhanced neuronal activity may negatively affect the demyelinated neurons themselves. Indeed, Na^2+^ and Ca^2+^ channel blocking agents have been shown to reduce damage to neurons in demyelinating lesions by inhibiting a neuronal cell membrane depolarizing mechanism (Brand-Schieber and Werner, 2004). Further damage to demyelinated neurons complicates their remyelination and this might explain why during cuprizone demyelination in mice, enhancing neuronal activity by behavioral intervention had no positive effects on remyelination directly after demyelination, but only as remyelination had commenced (Bacmeister et al., 2020). Future studies into neuronal-activity dependent myelin repair should take this precise timing into account, or may follow an alternative route in which activity-dependent pathways in OLs are stimulated directly instead of *via* neuronal activity (Jung et al., 2020).

Although emerging lines of evidence point to a role of neuronal activity in modulating oligodendroglia dynamics in lesions, few papers have investigated the signaling mechanisms that underlie the communication between electrically active axons and oligodendroglia during remyelination. Since the frequency of calcium transients is increased during remyelination (Battefeld et al., 2019), it is likely that OL calcium signaling pathways play a role, as occurs in adaptive myelination. However, whether intracellular calcium signaling has positive or negative effects on myelination under pathological conditions is unclear. For instance, ablation of the muscarinic M1 and M3 receptors that induce calcium responses in oligodendroglia enhances myelin repair, suggesting a negative role of muscarinic receptor-mediated calcium increases (Mei et al., 2016; Welliver et al., 2018). On the contrary, the removal of the inwardly rectifying calcium channel Cav1.2, which should reduce calcium activity, also impairs remyelination through decreases in OPC proliferation and maturation (Santiago González et al., 2017). While the activation of some receptors inducing calcium increases may have deleterious effects on myelin repair, specific intracellular calcium increases may be necessary to promote remyelination. In addition, it has been shown that in demyelinated lesions experimentally induced by the direct injection of gliotoxic agents, demyelinated axons make synaptic connections with OPCs that are positive for the glutamatergic synapse marker vGlut2, suggesting that axon-OPC synapses may play a role in remyelination (Etxeberria et al., 2010; Gautier et al., 2015; Sahel et al., 2015). Remarkably, an *in vitro* study has shown that magnetic field stimulation induces calcium influx in OPCs and enhances OPC differentiation and OL myelination (Prasad et al., 2017), suggesting that electrical stimulation *per se* may also induce direct effects on oligodendroglia in the absence of neuronal activity. Taken together, these findings suggest that complex mechanisms implicating calcium signals in OL lineage cells may be involved in neuronal activity-induced remyelination and that further research is necessary to unravel the associated signaling pathways.

## Methods of Neuronal Stimulation as Novel Therapies in MS

As emerging evidence suggests that increased neuronal activity can aid remyelination, it is worthwhile to examine studies in which MS patients with demyelinating lesions undergo neuronal stimulation. Deep brain stimulation is the only direct stimulation method that has been tried in MS patients. Several studies have shown that thalamic deep brain stimulation significantly reduces tremor and improves quality of life in MS patients (Hooper et al., 2002; Berk et al., 2002; Bittar et al., 2005; Thevathasan et al., 2011; Brandmeir et al., 2020). However, it is unlikely that the positive effects of deep brain stimulation on tremor symptoms rely on increased remyelination as the effects are seen quickly after stimulation. Nevertheless, it would be interesting to assess whether long treatment periods or different deep brain stimulation protocols influence local remyelination in patients. Clinical studies have also already explored non-invasive brain stimulation as an attractive therapeutic approach that is easily applicable in the clinic and, in some cases, can be applied at home (Gough et al., 2020). Although the mechanisms behind the effects of non-invasive brain stimulation are yet to be explained and probably result from mixed effects on neurons and glia, non-invasive brain stimulation in MS patients has been shown to reduce motor symptoms including spasticity, fatigue, neuropathic pain, tactile sensory problems, as well as urinary tract difficulties (Leocani et al., 2019). Different stimulation methods and various stimulation patterns have successfully been tried. Using repetitive (r)TMS for example, stimulation twice daily for 7 days has been shown to reduce spasticity when applied to the spine in MS patients (Nielsen et al., 1996) and high as well as low frequency (r)TMS improve spasticity when applied to the motor cortex for 2 weeks (Centonze et al., 2007). Notably, non-invasive brain stimulation appears to improve cognition in MS patients, a symptom that is not improved by current MS medication (DeLuca et al., 2020). tDCS applied to the dorsolateral PFC during 10 sessions of cognitive therapy enhanced training effects on executive functioning and attention in MS patients (Mattioli et al., 2016). The positive effects of tDCS applied during cognitive therapy were replicated in a second study for complex attention in MS patients (Charvet et al., 2018). Similar results were found using rTMS over the dorsolateral PFC which improved accuracy during a working memory task (Hulst et al., 2017). Collectively, these studies point to beneficial effects of non-invasive brain stimulation on alleviating diverse MS symptoms, sometimes in the long term, and are therefore a promising treatment avenue. However, whether non-invasive brain stimulation constitutes a strategy to enhance remyelination in MS is still unclear. In stroke patients, tDCS increases white matter integrity, often considered as a proxy for myelin integrity, which correlates with improved motor skills (Zheng and Schlaug, 2015). Enhanced white matter integrity after TMS has also been observed in aphasia patients (Lu et al., 2014). However, both studies used magnetic resonance imaging measures to assess white matter integrity and this technique is not sensitive enough to measure myelination directly, but rather represents a mix of myelin and axonal-related correlates. Similar studies investigating the effects of non-invasive brain stimulation on the state of demyelinating lesions in MS patients are still lacking. Therefore, further studies are necessary to find out whether the positive effects of non-invasive brain stimulation on MS symptomatology are indeed caused by enhanced remyelination.

Other than non-invasive brain stimulation, exercise and behavioral paradigms have also been shown to positively affect MS symptoms and their effects might be mediated by enhanced neuronal activity. For example, 8 weeks of high intensity exercise improve clinical outcome (Orban et al., 2019) and both exercise and cognitive training are effective in reducing cognitive impairments in MS patients (DeLuca et al., 2020). It has even been suggested that exercise should be prescribed as a medicine in early MS stages (Dalgas et al., 2019). As for non-invasive brain stimulation, the neurobiological mechanisms underlying the positive effects of exercise and cognitive training remain unknown. However, white matter integrity is increased in MS patients that have a high cardiorespiratory fitness (Prakash et al., 2010) indicating that remyelination might play a role, but future studies are still necessary to correlate symptom improvements with remyelination. An important challenge for future MS research studies will be to distinguish the benefits of non-invasive brain stimulation caused by an increased remyelination from those generated by improved neuronal regeneration, effects on astrocytes and microglia, or even effects on motivation and activeness of patients.

## Conclusion

Neuronal activity-dependent myelination can take place regardless of neuronal identity, but the effects of neuronal activity on oligodendroglial cells likely depend on the cumulative pattern of activity of a neuronal network. This means that both direct stimulation of neurons by for example optogenetics and indirect stimulation via non-invasive methods such as TMS and behavioral training can achieve *de novo* myelination in the healthy brain. The facts that neuronal activity can initiate myelination and that neuronal stimulation can be achieved relatively easily make activity-dependent myelination an attractive strategy for myelin repair in demyelinating disorders such as MS. Research into neuronal activity-dependent myelin repair is still scarce, however, the few *in vivo* preclinical studies that have been conducted suggest that enhanced neuronal activity in a demyelinated lesion indeed has the potential to improve remyelination (see Table 1 for an overview of preclinical and clinical studies applying direct and indirect neuronal stimulation and their effects on remyelination). Notably, as during adaptive myelination in the healthy brain, the pattern and frequency of the neuronal stimulation are important determinants of its effect as a single stimulation session had no lasting effects on remyelination, while repeated stimulation sessions significantly improved remyelination in the demyelinated mouse corpus callosum (Ortiz et al., 2019). In rodents, both direct stimulation by optogenetics and indirect stimulation by non-invasive brain stimulation or using behavioral training paradigms have positive effects on myelin repair after injury. Non-invasive brain stimulation and behavioral training can be readily applied in the clinic and, in MS patients, it has been shown that rTMS, tDCS and physical exercise can ameliorate disease symptomatology. However, it is yet to be determined whether this improvement of symptoms is caused by enhanced myelin repair after neuronal stimulation or whether the benefits of neuronal activity could be attributed to effects on the neurons themselves or on other brain cells (Table 1).

**TABLE 1:** Effects of direct and non-invasive neuronal stimulation on OPC proliferation, OPC differentiation, remyelination, and symptomatology in preclinical models and MS patients.

		Enhanced OPC proliferation in a lesion	Enhanced OPC differentiation in a lesion	Enhanced remyelination in a lesion	Improved symptomatology
Preclinical models	Direct stimulation	• Epidural electrical stimulation (Li et al., 2017) • Optogenetics (Ortiz et al., 2019)	• Epidural electrical stimulation (Li et al., 2017, 2020) • Optogenetics (Ortiz et al., 2019)	• Epidural electrical stimulation (Li et al., 2017, 2020) • Optogenetics (Ortiz et al., 2019)	• Epidural electrical stimulation (Li et al., 2017, 2020)
	Non-invasive stimulation	• Behavioral training (Bacmeister et al., 2020) • Voluntary exercise (Jensen et al., 2018)	• Behavioral training (Bacmeister et al., 2020) • Electromagnetic stimulation (Li et al., 2019)	• Behavioral training (Bacmeister et al., 2020) • Voluntary exercise (Jensen et al., 2018) • Electromagnetic stimulation (Sherafat et al., 2012) • Electromagnetic stimulation (Li et al., 2019) • Ultrasound burst stimulation (Olmstead et al., 2018)	• Electromagnetic stimulation (Li et al., 2019)
MS patients	Direct stimulation	NA	NA	NA	• Deep brain stimulation (Hooper et al., 2002; Berk et al., 2002; Bittar et al., 2005; Thevathasan et al., 2011)
	Non-invasive stimulation	NA	NA	NA	• High intensity exercise (Orban et al., 2019) • rTMS (Nielsen et al., 1996; Centonze et al., 2007; Hulst et al., 2017) • tDCS (Mattioli et al., 2016; Charvet et al., 2018)

**NA, Not Available.**

It is known that electrically active axons can communicate with oligodendroglia cells via synaptic and non-synaptic sites involving a number of receptors and messenger molecules that often lead to a depolarization of oligodendroglia and subsequent intracellular calcium signals. The nature of the communication from neurons to oligodendroglia and the frequency and amplitude of intracellular calcium increases in oligodendroglia are thought to determine the OL response to neuronal activity. Future studies need to reveal the molecular pathways involved in, and the calcium response characteristics of oligodendroglia to neuronal electrical activity.

In conclusion, the stimulation of neuronal activity is a promising strategy for myelin repair in demyelinating disorders such as MS and future research into the exact neurobiological mechanisms as well as clinical evaluation of changes in the brain after neuronal stimulation in patients will aid the development of this novel therapeutic approach to myelin repair.

## Author Contributions

DM and MCA wrote and corrected the manuscript. Both authors contributed to the article and approved the submitted version.

## Conflict of Interest

The authors declare that the research was conducted in the absence of any commercial or financial relationships that could be construed as a potential conflict of interest.
